# Supplementary material for: Experiencing a significant life event during the COVID-19 pandemic: The role of perceived control
Source: PLoS One. 2023 Jan 4;18(1):e0279820. doi: 10.1371/journal.pone.0279820 (PMC9812308; doi:10.1371/journal.pone.0279820)
Supplement: S1 File — (DOCX) [file pone.0279820.s001.docx]

**Supporting Information**

**S1 Table. German and English Wording of the Items.**

| German Item | English Translation | Scale |
| --- | --- | --- |
| Umzug in ein anderes Land | Relocation to another country | Life event |
| Umzug in eine andere Stadt/einen anderen Wohnort | Relocation to another city / place of residence | Life event |
| Wohnungswechsel am gleichen Wohnort | Change of residence in the same city | Life event |
| Längerer Aufenthalt im Ausland | Longer stay abroad | Life event |
| Eine neue romantische Beziehung eingegangen (von mindestens einem Monat Dauer) | Entered a new romantic relationship (of at least a month) | Life event |
| Heirat/ eingetragene Partnerschaft | Marriage / registered partnership | Life event |
| Geburt eines Kindes | Birth of a child | Life event |
| Trennung oder Scheidung | Separation or divorce | Life event |
| Eine wichtige soziale Beziehung beendet (ausser Trennung oder Scheidung) | Ended an important social relationship (other than separation or divorce) | Life event |
| Eigene schwerwiegende Krankheit oder Verletzung | Own serious illness or injury | Life event |
| Schwerwiegende Krankheit oder Verletzung des Partners/der Partnerin | Serious illness or injury of the partner | Life event |
| Schwerwiegende Krankheit oder Verletzung eines nahen Familienmitgliedes oder Freundes/Freundin | Serious illness or injury of a close family member or a friend | Life event |
| Tod des Partners/der Partnerin | Loss of the partner | Life event |
| Tod eines nahen Familienmitgliedes oder Freundes/Freundin | Loss of a close family member or a friend | Life event |
| Längerer Aufenthalt im Spital/Psychiatrie/Gefängnis oder vergleichbarer Institution | Long stay in hospital / psychiatry / prison or comparable institution | Life event |
| Beförderung im Job | Job promotion | Life event |
| Stellenwechsel | Job change | Life event |
| Arbeitslosigkeit | Unemployment | Life event |
| Verrentung | Retirement | Life event |
| Wie haben Sie das Ereignis erlebt?  Positiv  Negativ  Erwünscht  Unerwünscht  Belastend  Bereichernd | How did you experience the event?  Positive  Negative  Desirable  Undesirable  Burdening  Enriching | Life-event experience  Life-event experience  Life-event experience  Life-event experience |
| Wie viel Kontrolle hatten Sie über die verschiedenen Phasen des Ereignisses? | How much control did you have over the different stages of the event? | Perceived control |
| Wie viel Kontrolle hatten Sie über das Eintreten des Ereignisses? | How much control did you have over the occurrence of the event? | Perceived control |
| Wie viel Kontrolle hatten Sie über den Verlauf des Ereignisses?  Wie viel Kontrolle hatten Sie über den Ausgang des Ereignisses? | How much control did you have over the course of the event?  How much control did you have over the outcome of the event? | Perceived control  Perceived control |
| Inwieweit wurde das Ereignis von der Corona-Situation beeinflusst? | To what extent was the event influenced by the corona situation? |  |
| Stärke Einfluss | Influence intensity | COVID-19 life event influence |
| Positiver Einfluss | Positive influence | COVID-19 life event influence |
| Negativer Einfluss | Negative influence | COVID-19 life event influence |
| Inwieweit wurde Ihr Erleben des Ereignisses von der Corona-Situation beeinflusst? | To what extent was your experience of the event influenced by the corona situation? |  |
| Stärke Einfluss | Influence intensity | COVID-19 life event experience influence |
| Positiver Einfluss | Positive influence | COVID-19 life event experience influence |
| Negativer Einfluss | Negative influence | COVID-19 life event experience influence |

*Note.* English translation is based on the German version of the item.

**S2 Table. Descriptive Statistics for all 19 Individual Life Events.**

|  |  | Age | | Gender | | | | Self-reported COVID-19 influence intensity | | Self-reported COVID-19 positive influence | | Self-reported COVID-19 negative influence | | Life-event Experience | | Perceived Control | |
| --- | --- | --- | --- | --- | --- | --- | --- | --- | --- | --- | --- | --- | --- | --- | --- | --- | --- |
| Life events | n | M SD | | | m f | | M SD | | M SD | | M SD | | M SD | | M SD | |  |
| Relocation to another country | 7 | 38.71 18.16 | | | 71.4 28.6 | | 4.86 1.73 | | 3.14 1.80 | | 5.43 1.30 | | 5.36 1.42 | | 5.38 1.06 | |  |
| Relocation to another city / place of residence | 46 | 34.35 14.81 | | | 56.5 43.5 | | 4.04 2.29 | | 2.79 1.68 | | 3.91 1.94 | | 5.29 1.19 | | 5.54 1.15 | |  |
| Change of residence in the same city | 54 | 41.91 16.19 | | | 57.4 40.7 | | 3.80 1.97 | | 3.34 1.55 | | 3.61 1.78 | | 5.21 1.33 | | 5.40 1.11 | |  |
| Longer stay abroad | 4 | 59.00 12.44 | | | 25.0 75.0 | | 3.75 0.65 | | 3.63 1.11 | | 2.88 1.49 | | 6.21 1.27 | | 4.83 1.60 | |  |
| Marriage/registered partnership | 25 | 36.32 9.89 | | | 56.0 44.0 | | 4.52 2.03 | | 3.48 1.66 | | 3.92 1.96 | | 6.51 0.81 | | 5.99 1.09 | |  |
| Entered a new romantic relationship | 39 | 41.33 15.56 | | | 53.8 43.6 | | 4.15 1.97 | | 3.58 1.78 | | 3.65 1.88 | | 6.38 0.78 | | 4.56 1.51 | |  |
| Birth of a child | 49 | 38.68 10.19 | | | 44.9 55.1 | | 4.87 1.94 | | 3.26 1.83 | | 4.11 1.79 | | 6.24 0.72 | | 4.10 1.91 | |  |
| Separation or divorce | 19 | 32.58 12.18 | | | 57.9 42.1 | | 4.13 1.79 | | 3.11 1.29 | | 4.29 1.38 | | 2.50 1.27 | | 4.09 1.73 | |  |
| Ended an important social relationship | 35 | 39.43 15.63 | | | 54.3 45.7 | | 3.33 2.06 | | 2.49 1.78 | | 3.57 1.94 | | 2.96 1.90 | | 3.73 1.92 | |  |
| Own serious illness or injury | 67 | 56.79 15.29 | | | 65.7 34.3 | | 3.46 2.34 | | 2.46 1.59 | | 3.69 2.23 | | 1.73 1.14 | | 2.75 1.49 | |  |
| Serious illness or injury of the partner | 41 | 60.71 10.89 | | | 46.3 53.7 | | 4.60 2.03 | | 1.91 1.78 | | 4.63 1.81 | | 1.69 1.15 | | 2.34 1.52 | |  |
| Serious illness or injury of a close family member or a friend | 71 | 51.24 14.40 | | | 43.7 56.3 | | 3.94 2.37 | | 1.97 1.21 | | 4.30 2.20 | | 1.44 0.69 | | 1.66 1.06 | |  |
| Loss of the partner | 9 | | 66.67 7.81 | | | 11.1 88.9 | 3.39 2.80 | | 1.78 1.56 | | 4.44 2.71 | | 1.59 1.12 | | 1.67 1.09 | |  |
| Loss of a close family member or a friend | 175 | | 53.41 14.54 | | | 48.6 51.4 | 3.54 2.37 | | 1.87 1.35 | | 3.61 2.31 | | 1.57 0.83 | | 1.66 1.33 | |  |
| Long stay in hospital / psychiatry / prison or comparable institution | 5 | | 41.20 7.85 | | | 40.0 60.0 | 4.90 2.92 | | 1.40 0.65 | | 4.90 2.92 | | 1.37 0.51 | | 2.93 2.35 | |  |
| Job promotion | 29 | | 40.00 11.90 | | | 72.4 27.6 | 3.05 1.74 | | 3.45 1.89 | | 2.88 1.75 | | 6.06 0.96 | | 5.03 1.20 | |  |
| Job change | 73 | | 39.12 10.45 | | | 43.8 56.2 | 3.66 2.08 | | 3.49 1.85 | | 3.15 1.83 | | 5.43 1.54 | | 4.77 1.55 | |  |
| Unemployment | 75 | | 44.71 13.27 | | | 62.7 37.3 | 5.55 1.73 | | 2.27 1.66 | | 5.48 1.81 | | 1.98 1.41 | | 2.00 1.43 | |  |
| Retirement | 38 | | 62.00 5.56 | | | 60.5 39.5 | 3.03 2.39 | | 3.11 1.95 | | 2.67 1.89 | | 5.96 1.13 | | 4.45 2.41 | |  |
| Other life event | 21 | | 55.67 17.45 | | | 42.9 57.1 | 5.45 2.24 | | 2.45 1.56 | | 4.81 2.11 | | 3.42 2.02 | | 2.76 1.76 | |  |

*Note*. m = male, f = female for gender in percent.

**S3 Table. Results for Individual Life Events with the Life-Event Experience as the Outcome Variable Without Gender and Education as Control Variables.**

|  |  | β | p | 95% CI  LL UL | R^2^ | ΔR² |
| --- | --- | --- | --- | --- | --- | --- |
| Relocation  Model 1    Model 2    Model 3 | Positive influence  Age  Negative influence  Age  Perceived control  Age | -.13  -.04  -.27  -.06  **.34**  -.03 | .19  .70  .12  .54  **<.001**  .73 | -.250 .050  -.018 .012  -.410 .046  -.020 .010  .**183 .599**  -.017 .012 | .016  .020  .118 | .001  .003  .011 |
| Birth of a child  Model 1    Model 2    Model 3 | Positive influence  Age  Negative influence  Age  Perceived control  Age | .11  .25  **-.36**  .22  **.51**  **.54** | .48  .10  **.04**  .12  **.003**  **.002** | -.077 .161  -.003 .039  **-.277 -.010**  -.004 .035  **.070 .317**  **.015 .061** | .003  .092  .222 | .060  .049  .184 |
| Own serious illness or injury  Model 1    Model 2    Model 3 | Positive influence  Age  Negative influence  Age  Perceived control  Age | .07  .17  -.05  .17  **.27**  .14 | .55  .17  .83  .18  **.03**  .23 | -.123 .226  -.006 .031  -.236 .189  -.006 .030  **.023 .388**  -.007 .028 | .004  .001  .101 | .028  .027  .020 |
| Serious illness or injury of a loved one  Model 1    Model 2    Model 3 | Positive influence  Age  Negative influence  Age  Perceived control  Age | **.25**  -.07  -.31  -.13  **.39**  -.12 | **.01**  .49  .09  .18  **<.001**  **.18** | **.046 .327**  **-.016 .008**  -.283 .019  -.020 .004  .**151 .391**  -.019 .004 | .069  .024  .168 | .004  .017  .014 |
| Loss of a loved one  Model 1    Model 2    Model 3 | Positive influence  Age  Negative influence  Age  Perceived control  Age | **.30**  .13  **-.52**  **.12**  **.27**  .11 | **<.001**  **.08**  **<.001**  **.10**  **<.001**  .13 | **.100 .278**  -.001 .015  **-.292 -.084**  **.**098 -.001    .084 .263  -.002 .014 | .083  .063  .087 | .016  .014  .011 |
| Job change  Model 1    Model 2    Model 3 | Positive influence  Age  Negative influence  Age  Perceived control  Age | **.26**  .01  **-.59**  .09  **.56**  -.003 | .**04**  .93  **<.001**  .41  **<.001**  .98 | .016 .422  -.033 .036  -.736 -.263  -.019 .045  .358 .752  -.030 .029 | .063  .196  .312 | .000  .008  .000 |
| Unemployment  Model 1    Model 2    Model 3 | Positive influence  Age  Negative influence  Age  Perceived control  Age | **.43**  .06  **-.62**  .03  **.49**  .01 | **<.001**  .57  **.007**  .79  **<.001**  .92 | .183 .545  -.016 .029  -.822 -.136  -.020 .027  .278 .687  -.021 .023 | **.168**  **.165**  **.239** | **.003**  .001  .000 |

*Note*. Controlled for influence intensity. CI = confidence interval. Significant results (*p* < .05) are in bold.

**S4 Table**. **Results for Individual Life Events with the Perceived Control as the Outcome Variable Without Gender and Education as Control Variables.**

|  |  | β | p | 95% CI  LL UL | R^2^ | ΔR² |
| --- | --- | --- | --- | --- | --- | --- |
| Relocation  Model 1    Model 2 | Positive influence  Age  Negative influence  Age | .04  .04  -.02  .03 | .72  .72  .90  .75 | -.112 .161  -.011 .016  -.221 .194  -.012 .016 | .001  .000 | .001  .001 |
| Birth of a child  Model 1    Model 2 | Positive influence  Age  Negative influence  Age | .38  -.52  **-.34**  -.60 | .001  <.001  **.01**  **<.001** | .166 .629  -.138 -.056  **-.651 -.082**  -.156 -.071 | .237  .076 | .256  .365 |
| Own serious illness or injury  Model 1    Model 2 | Positive influence  Age  Negative influence  Age | .07  .11  -.28  .11 | .56  .37  .18  .40 | -.164 .300  -.013 .035  -.467 .091  -.014 .034 | .005  .028 | .013  .011 |
| Serious illness or injury of a loved one  Model 1      Model 2 | Positive influence  Age  Negative influence  Age | **.15**  .03  -.14  -.01 | **.14**  .77  .44  .94 | **-.050 .368**  **-.015 .020**  -.309 .135  -.018 .017 | .020  .005 | .001  .000 |
| Loss of a loved one  Model 1    Model 2 | Positive influence  Age  Negative influence  Age | **.19**  .04  **-.17**  **.03** | **.01**  **.62**  **.26**  **.69** | .041 .332  -.010 .017    **-.266 .073**  **-.011** .016 | .034  .007 | .001  .001 |
| Job change  Model 1    Model 2 | Positive influence  Age  Negative influence  Age | **.30**  .03  **-.17**  .07 | .**02**  .79  **.27**  .59 | .048 .452  -.030 .039  -.411 .118  -.026 .045 | .082  .015 | .001  .004 |
| Unemployment  Model 1    Model 2 | Positive influence  Age  Negative influence  Age | **.41**  **-.03**  **-.56**  -.06 | **<.001**  .79  **.01**  .58 | .175 .536  -.025 .019  -.785 -.100  -.030 .017 | **.170**  **.085** | **.001**  .003 |

*Note.* Controlled for influence intensity. CI = confidence interval*.* Significant results (*p* < .05) are in bold.

**S5 Table. Mediation Analyses’ Results for Perceived Control as a Mediator Between Self-Reported Influence of the Pandemic on the Life Event and Life-Event Experience Across Individual Significant Life Events.**

|  |  | Path | Coefficient | SE | t | p | | 95% CI  LL UL |
| --- | --- | --- | --- | --- | --- | --- | --- | --- |
| Relocation | PI  NI | c  a  b  direct effect (c’)  indirect effect  c  a  b  direct effect (c’)  indirect effect | -.11  .004  **.39**  --  --  -.19  -.02  .**39**  --  -- | .07  .07  .**10**  .07  .03  .11  .10  **.10**  .11  .05 | -1.45  0.06  **3.82**  -1.45  --  -1.64  -0.24  **3.78**  -1.65  -- | .15  .95  **<.001**  .15  --  .10  .81  **<.001**  .10  -- | -.249 .039  -.134 .142  **.189 .599**  -.249 .039  -.050 .066  -.415 .040  -.231 .181  **.185 .593**  -.392 .036  -.091 .096 | |
| Birth of a child | PI  NI | c  a  b  direct effect (c’)  indirect effect  c  a  b  direct effect (c’)  indirect effect | .02  .**49**  .09  --  --  **-.15**  -.30  .06  --  -- | .06  **.15**  .06  .07  .04  **.07**  .18  .06  .07  .02 | 0.33  **3.29**  1.45  -0.35  --  **-2.16**  -1.61  1.02  -1.86  -- | .74  **.002**  .15  .73  --  **.04**  .11  .31  .07  -- | -.109 .151  **.190 .790**  -.037 .224  -.168 .119  -.008 .159  **-.294 -.010**  -.669 .074  -.057 .174  -.280 .011  -.076 .018 | |
| Own serious illness or injury | PI  NI | c  a  b  direct effect (c’)  indirect effect  c  a  b  direct effect (c’)  indirect effect | .04  .05  **.20**  --  --  -.03  -.16  **.20**  --  -- | .09  .12  **.09**  .09  .03  .11  .14  **.09**  .11  .03 | 0.51  0.42  **2.09**  0.41  --  -0.31  -1.15  **2.09**  -0.01  -- | .61  .67  .**04**  .69  --  .76  .25  **.04**  .99  -- | -.131 .220  -.183 .281  **.009 .383**  -.137 .206  -.038 .085  -.251 .184  -.447 .120  **.009 .387**  -.215 .213  -.097 .031 | |
| Serious illness or injury of a loved one | PI  NI | c  a  b  direct effect (c’)  indirect effect  c  a  b  direct effect (c’)  indirect effect | **.20**  .15  **.25**  --  --  -.13  -.09  **.26**  --  -- | **.07**  .10  **.06**  **.07**  .03  .08  .11  **.06**  .07  .03 | **2.81**  1.43  **4.15**  **2.41**  --  -1.68  -0.79  **4.32**  -1.48  -- | .**006**  .16  **<.001**  **.02**  --  .10  .43  **<.001**  .14  -- | **.057 .334**  -.058 .355  **.131 .370**  **.028 .289**  -.017 .110  -.280 .023  -.311 .134  **.143 .385**  -.246 .036  -.098 .036 | |
| Loss of a loved one | PI  NI | c  a  b  direct effect (c’)  indirect effect  c  a  b  direct effect (c’)  indirect effect | .**17**  **.17**  **.14**  --  --  **-.17**  -.10  **.15**  --  -- | .**04**  **.07**  **.04**  **.04**  .02  **.05**  .09  **.04**  **.05**  .02 | **3.80**  **2.33**  **3.10**  **3.30**  --  **-3.18**  -1.16  **3.43**  **-2.96**  -- | **<.001**  **.02**  **.002**  **.001**  --  **.002**  .25  **<.001**  .**004**  -- | **.082 .258**  **.026 .315**  **.050 .225**  **.059 .234**  -.001 .060  **-.269 -.063**  -.269 .070  **.064 .239**  **-.252 -.050**  -.066 .016 | |
| Job change | PI  NI | c  a  b  direct effect (c’)  indirect effect  c  a  b  direct effect (c’)  indirect effect | **.23**  **.25**  **.51**  --  --  **-.47**  -.14  **.50**  --  -- | **.10**  **.10**  **.10**  .09  .05  **.11**  .13  **.09**  **.10**  .08 | **2.31**  **2.47**  **4.92**  1.15  --  **-4.14**  -1.05  **5.52**  **-4.22**  -- | **.02**  **.02**  **<.001**  .25  --  **<.001**  . 30  **<.001**  **<.001**  -- | **.031 .424**  **.047 .445**  **.302 .716**  -.075 .278  .019 .235  **-.702 -.245**  -.393 .122  **.316 .675**  **-.599 -.214**  -.222 .071 | |
| Unemployment  PI  NI |  | c  a  b  direct effect (c’)  indirect effect  c  a  b  direct effect (c’)  indirect effect | **.37**  **.36**  **.32**  --  --  **-.52**  -.34  **.41**  --  -- | **.09**  **.08**  **.12**  **.09**  .06  **.18**  .18  **.11**  **.17**  .08 | **4.16**  **4.32**  **2.68**  **2.53**  --  **-2.88**  -1.88  **3.72**  **-2.24**  -- | **<.001**  **<.001**  **.01**  **.01**  --  **.01**  .06  **<.001**  **.03**  -- | **.192 .545**  **.195 .530**  **.083 .564**  **.061 .441**  .001 .240  **-.883 -.160**  -.692 .021  **.192 .636**  **-.723 -.042**  -.326 -.001 | |

*Note.* PI = self-reported positive COVID-19 influence, NI = self-reported negative COVID-19 influence. SE = standard error; CI = confidence interval. Self-reported COVID-19 influence intensity, gender and education were considered covariates. Significant results (*p* < .05) are in bold.

**S6 Table. Mediation Analyses’ Results for Perceived Control as a Mediator Between Self-Reported Influence of the Pandemic on the Life Event and Life-Event Experience Across Individual Significant Life Events Without Gender and Education as Covariates.**

|  |  | Path | Coefficient | SE | t | p | | 95% CI  LL UL |
| --- | --- | --- | --- | --- | --- | --- | --- | --- |
| Relocation | PI  NI | c  a  b  direct effect (c’)  indirect effect  c  a  b  direct effect (c’)  indirect effect | -.11  .02  **.39**  --  --  -.17  -.02  .**38**  --  -- | .07  .07  .**10**  .07  .03  .11  .10  **.10**  .11  .05 | -1.53  0.35  **3.84**  -1.53  --  -1.51  -0.19  **3.76**  -1.53  -- | .13  .73  **<.001**  .13  --  .13  .85  **<.001**  .13  -- | -.249 .032  -.112 .159  **.188 .591**  -.249 .032  -.045 .073  -.395 .054  -.223 .185  **.180 .583**  -.375 .048  -.087 .048 | |
| Birth of a child | PI  NI | c  a  b  direct effect (c’)  indirect effect  c  a  b  direct effect (c’)  indirect effect | .02  .**52**  .08  --  --  **-.15**  -.35  .04  --  -- | .06  **.14**  .06  .07  .04  **.07**  .18  .06  .07  .02 | 0.35  **3.78**  1.33  -0.34  --  **-2.17**  -1.95  0.81  -1.85  -- | .73  **<.001**  .19  .73  --  **.04**  .06  .42  .07  -- | -.098 .139  **.241 .790**  -.044 .211  -.157 .112  -.016 .134  **-.281 -.010**  -.713 .011  -.067 .156  -.272 .012  -.073 .023 | |
| Own serious illness or injury | PI  NI | c  a  b  direct effect (c’)  indirect effect  c  a  b  direct effect (c’)  indirect effect | .05  .06  **.20**  --  --  -.03  -.19  **.21**  --  -- | .09  .12  **.09**  .09  .03  .11  .14  **.09**  .11  .03 | 0.53  0.55  **2.21**  0.40  --  -0.25  -1.37  **2.23**  0.12  -- | .60  .58  .**03**  .69  --  .80  .18  **.03**  .91  -- | -.129 .223  -.168 .295  **.019 .387**  -.137 .205  -.031 .093  -.241 .187  -.470 .087  **.021 .394**  -.198 .223  -.105 .027 | |
| Serious illness or injury of a loved one | PI  NI | c  a  b  direct effect (c’)  indirect effect  c  a  b  direct effect (c’)  indirect effect | **.20**  .15  **.25**  --  --  -.12  -.09  **.26**  --  -- | **.07**  .10  **.06**  **.07**  .03  .08  .11  **.06**  .07  .03 | **2.85**  1.48  **4.16**  **2.44**  --  -1.64  -0.77  **4.35**  -1.44  -- | .**005**  .14  **<.001**  **.02**  --  .10  .44  **<.001**  .15  -- | **.060 .334**  -.051 .356  **.131 .370**  **.030 .288**  -.018 .113  -.276 .026  -.307 .134  **.144 .385**  -.243 .038  -.098 .035 | |
| Loss of a loved one | PI  NI | c  a  b  direct effect (c’)  indirect effect  c  a  b  direct effect (c’)  indirect effect | .**18**  **.18**  **.15**  --  --  **-.18**  -.09  **.16**  --  -- | .**05**  **.07**  **.04**  **.05**  .02  **.05**  .09  **.04**  **.05**  .02 | **4.05**  **2.51**  **3.27**  **3.48**  --  **-3.48**  -1.11  **3.68**  **-3.29**  -- | **<.001**  **.01**  **.001**  **<.001**  --  **<.001**  .27  **<.001**  .**001**  -- | **.094 .274**  **.039 .329**  **.058 .235**  **.068 .246**  .000 .069  **-.288 -.080**  -.264 .074  **.076 .251**  **-.270 -.067**  -.071 .020 | |
| Job change | PI  NI | c  a  b  direct effect (c’)  indirect effect  c  a  b  direct effect (c’)  indirect effect | **.22**  **.25**  **.53**  --  --  **-.49**  -.14  **.51**  --  -- | **.10**  **.10**  **.10**  .09  .05  **.12**  .13  **.09**  **.10**  .08 | **2.18**  **2.51**  **5.19**  0.95  --  **-4.15**  -1.04  **5.80**  **-4.27**  -- | **.03**  **.01**  **<.001**  .34  --  **<.001**  .30  **<.001**  **<.001**  -- | **.019 .421**  **.051 .452**  **.327 .736**  -.094 .265  .030 .247  **-.719 -.252**  -.396 .125  **.337 .690**  **-.610 -.221**  -.250 .074 | |
| Unemployment  PI  NI |  | c  a  b  direct effect (c’)  indirect effect  c  a  b  direct effect (c’)  indirect effect | **.35**  **.36**  **.31**  --  --  **-.47**  -.46  **.38**  --  -- | **.09**  **.09**  **.11**  **.09**  .06  **.17**  .17  **.11**  **.16**  .08 | **3.99**  **4.11**  **2.76**  **2.55**  --  **-2.80**  -2.75  **3.46**  **-1.79**  -- | **<.001**  **<.001**  **.007**  **.01**  --  **.007**  .008  **.001**  **.08**  -- | **.177 .530**  **.186 .535**  **.087 .541**  **.052 .427**  .003 .229  **-.805 -.135**  -.795 -.126  **.161 .599**  **-.623 .033**  -.338 -.047 | |

*Note.* PI = self-reported positive COVID-19 influence, NI = self-reported negative COVID-19 influence. SE = standard error; CI = confidence interval. Self-report COVID-19 influence intensity was considered a covariate. Significant results (*p* < .05) are in bold.
