# Supplementary material for: Experiencing a significant life event during the COVID-19 pandemic: The role of perceived control
Source: PLoS One. 2023 Jan 4;18(1):e0279820. doi: 10.1371/journal.pone.0279820 (PMC9812308; doi:10.1371/journal.pone.0279820)
Supplement: Attachment — Submitted filename: PONE-D-22-01404 Experiencing a Significant Life Event During the COVID-19 Pandemic- The Role of Perceived Control PLOS ONE_Cory Counts.docx [file pone.0279820.s002.docx]

Thank you for taking the time to conduct the following research. I enjoyed reading your manuscript, “Experiencing a significant life event during the COVID -19 pandemic: The role of perceived control”. I thought that the overall idea was interesting and has important implications for better understanding how major global stressors such as the COVID-19 pandemic may impact an individual’s perception of major life events contingent upon perceived control over one’s life.

Below are some comments and changes I personally recommend to be considered for publication.

**Comments and concerns:**

Did your lab collect data on self-reported anxiety and/or depression? As mentioned, in sentence 94-95, research had found the pandemic had a deleterious effect on adult mental health. The two variables would seemingly have important implications for individual perception of perceived control and the valence of life events. If you did collect measures of depression and/or anxiety, I would consider controlling for these two variables and rerunning your analyses. If not, I think adding a note of this in the limitations would be worthwhile.

What is the justification for not controlling for gender?

Did you collect data on socioeconomic status? Differences in income would seemingly be important to understand in how they may explain the relationship between perception of positive or negative influence of the pandemic on major life events and perceived self-control. Income is an additional variable I would consider controlling for if you did collect data for this measure.

It may be worth noting that the following results may look different based upon geographical location. The following results may differ in countries where social systems such as universal healthcare and economic aid during the pandemic differed.

In sentence 57 you mention how ongoing stressors may lead to more negative responses to significant life events, although stress was not mentioned or controlled for in analyses. Did you include a measure of stress in your data collection process?

**Manuscript grammatical changes**

Sentence 54-55: COVID-19 is a pandemic respiratory disease [5, 6] that led many countries to order lockdowns that reduce social contacts.

*I think you can delete pandemic from this sentence, as it was mentioned in the previous sentence.*

Sentence 69: The following sentence could be made better with grammatical changes. “We argue that an important factor that is associated with well-being in the pandemic in general—and in life events that occur during the pandemic in particular—is perceived control.”

*Maybe instead you could say, “We argue that an important factor associated with general well-being during the pandemic, and with particular life events that occur during the pandemic, is perceived control.*

Sentence 76: “alongside the significant life event” – *perhaps change to a significant life event*

Sentence 135: “Data were collected online between June 23 and July 8, 2020.” -- *Change were to was, or data were to data points were.*

Sentence 205: “fit according to Cohen.” -- *Perhaps specify Cohen’s d*.

Sentence 215: “life-event experience, there were a significant association”. – *There were significant associations*
