# Supplementary material for: Experiencing a significant life event during the COVID-19 pandemic: The role of perceived control
Source: PLoS One. 2023 Jan 4;18(1):e0279820. doi: 10.1371/journal.pone.0279820 (PMC9812308; doi:10.1371/journal.pone.0279820)
Supplement: Attachment — Submitted filename: Response to reviewers.docx [file pone.0279820.s003.docx]

Dear Dr. John-Henderson,

Thank you for the opportunity to revise our manuscript and for your and the reviewers’ helpful comments and suggestions. We believe that the changes inspired by the review process strengthened the manuscript and its message. Bellow, we respond to all questions raised by you and the reviewers. Changes in the manuscript are marked in red. Thank you for considering our research for publication.

Sincerely,

The Authors

**Editor’s comment:**

Thank you for submitting your manuscript to PLOS ONE. After careful consideration, we feel that it has merit but does not fully meet PLOS ONE’s publication criteria as it currently stands. Therefore, we invite you to submit a revised version of the manuscript that addresses the points raised during the review process.

While the reviewers and I agree that the topic of the manuscript is interesting and important, there are several major concerns that need to be addressed. Please make sure to address all of the major and minor concerns outlined by each reviewer.

**Response:** Thank for the overall positive evaluation of our manuscript.

**Reviewer 1 Comment 1:**

Thank you for your very interesting study. However, I recommend studying basic academic writing and research methodology before resubmission.

**Response:** Thank you for the positive feedback. We hope that the changes made have further strengthened the manuscript.

**Reviewer 1 Comment 2:**

In Introduction, please systematically summarize what is known and what is not known about perceived control in COVID-19 pandemic.

**Response:** Thank you for this suggestion. There are very few studies that investigated perceived control during the pandemic. We searched for any more recent (published after our submission) or previously overlooked papers, and we included them in the manuscript) (L77-84). Other studies we found addressed more specific issues about the pandemic that included perceived control as a variable – we included these in the manuscript as well (L78-84).

**Reviewer 1 Comment 3:**

It is unclear if you assume perceived control as a predictor or outcome or both (?).
It is better to draw a conceptual framework, based on theories.

**Response:** Perceived control is both predictor and outcome, as the hypotheses represent mediation with separate regression analyses (L105-113). In other words, we hypothesize that the self-reported influence of the pandemic on the life event predicts perceived control, which in turn predicts life-event experience. For clarification we added L81-84 and in the abstract L35-36. For illustration, we have included Figure S1 as Figure 2 in the manuscript (L303) and mentioned plots A-F from Figure 1 where relevant results were reported in the text. We have also changed the order of Hypotheses 2 and 3 for better understanding, and therefore also changed the order of plots in Figure 1.

**Reviewer 1 Comment 4:**

Is description written in L117-128 from your previous study or this study? If it is from this study, please include it in Results.

**Response:** We apologize for the confusion. The section describes the analytical approach of the present study (L114-126), and the outcomes are included in the Results H1-H3.

**Reviewer 1 Comment 5:**

L130
Please define age-heterogenous sample and how you accessed them.

**Response:** We now report the distribution of the sample across decades (L201-202). We have also added more details about the recruitment of participants (L131-135).

**Reviewer 1 Comment 6:**

Please explain more about online recruitment service. Did you use e-mail or some social media? It would not be easy to approach older people, and I wonder how you did.

**Response:** We have added more information about the recruitment of participants (L131-135).

**Reviewer 1 Comment 7:**

L139-145
I think this part should also be written in Results.

**Response:** We have moved this paragraph to Results (“Descriptive analyses”). Table 1 and 2 are now also listed in Results (L219 and L221).

**Reviewer 1 Comment 8:**

L146- Measures
Please describe more about scales, including reliability and validity with references if applicable.

**Response:** Thank you for your suggestion. We included the Cronbach’s Alpha for all scales (L165, L171, L177, L178). As the scales were self-developed, we do not report any validity references.

**Reviewer 1 Comment 9:**

P173-
Explanation of Tables should be included in Results.

**Response:** Thank you for pointing this out. We have added a more detailed description for Table 1 (L207-217). The Table 2 is self-explanatory and the Tables 3 and 4 have in our opinion already been explained (L245-251; L261-264; 275-281).

**Reviewer 1 Comment 10:**

In Method, you need to explain Analysis and Ethical consideration. Have you obtained permission from Institutional Review Board? How did you explain and obtained agreement from participants?

**Response:** The analyses were explained at the beginning of the Results – however, we have transferred this part to Methods (L180-184). We received approval from the Ethics Committee (this information can be found on L130-131). At your suggestion, we have added more information about informed consent (L136-138).

**Reviewer 1 Comment 11:**

What was the significant level you set? There are many analyses. It is better to set lower significant level to decrease alpha error.

**Response:** We set the significance level to .05 and the information is now added in the section where we report the power analysis (L190). We did not adjust the significance level because this increases type II error and too much emphasis is placed on significance levels rather than effect sizes and explained variance (Nakagawa, 2004). We prefer to report the latter rather than adjust the significance level. In addition, the Bonferroni correction (.006 for the number of our analyses) would not change the findings because of the already highly significant results. We provide reasons in the Discussion (L341-345) for not making corrections to account for multiple comparisons.

**Reviewer 1 Comment 12:**

Did you use linear regression? Have you checked the distribution (normality) of outcome variables?

**Response:** Yes, we conducted linear regression analyses. As for the distribution of outcome variables, according to the literature, the use of linear regression analyses is legitimate, especially for large samples, despite the violation of the normality rule (of residuals). Furthermore, multiple linear regression is sufficiently robust to the assumption of a normal distribution in most cases (e.g., Li et al., 2012; Schmidt & Finan, 2018). However, we offer to indicate the normal distribution in the supplemental material if this seems necessary.

**Reviewer 1 Comment 13:**

L182-195
This part should be written in Methods.

**Response:** We transferred this part to Methods (L180-184).

**Reviewer 1 Comment 14:**

L193-195
You also have to consider alpha error. Please also exactly report the effect size, analysis, one tail or two tail, which you set in G*Power.

**Response:** Thank you for pointing this out. We have included this information in the manuscript (L185-193).

**Reviewer 1 Comment 15:**

Please indicate number of participants in Table 1 and 2.

**Response:** We have added the number of participants in Table 1 (it was already included in Table 2).

**Reviewer 1 Comment 16:**

In Table 2, total number is 667, which is smaller than 882. If 215 participants answered others, it should be included in the Table.

**Response:** The total number is 667 because, according to the power analysis, some single life events could not be analyzed. To avoid confusion, we have added another sentence in the text (L192-193) and in the table legend (L222) to clarify this.

**Reviewer 1 Comment 17:**

In Result, we usually do not cite references. You do not need summary of results and conclusion in Results. It should be written in Discussion and Conclusion.

**Response:** We agree that the summary in the Discussion is sufficient and have deleted the summaries in the Results. We have also moved the information on the statistical program used (L183) and the Cohen goodness-of-fit indices (L193-196) with the corresponding references to the Methods to avoid references in the results.

**Reviewer 1 Comment 18:**

The contents and form of Table 3 and 4 seem also to be unusual. It is unusual to report only t, βand p in this form.

**Response:** Thank you for pointing this out. The table format, as well as the results in the text, have now been changed to include the estimate, confidence intervals, and *p*, and exclude the t-values.

**Reviewer 1 Comment 19:**

Figure 1 is unclear and hard to see.

**Response:** We have changed the formatting according to the journal’s requirements using the online tool suggested by the journal.

**Reviewer 1 Comment 20:**

L297-8
“The findings of the present study demonstrate that the pandemic affects how we experience significant life events.”
It is hard to say that your study indicated that pandemic affects experience of life-events since you did not compare to the situation without pandemic.

**Response:** The quote mentioned was used because the items in the study were specifically formulated about the pandemic. However, we agree that the conclusion was too strong and have weakened the wording. The sentence now reads, “The results of the present study suggest that the pandemic might affect the way we experience significant life events” (L313-314).

**Reviewer 2 Comment 1:**

I have made several comments on measures or outcomes that are mentioned in the introduction to be related to the author's results (e.g., mental health, stress) but not utilized in analyses.

The authors utilized appropriate statistical models for their hypotheses and included exploratory analyses that further strengthen their overall hypotheses.

I have made comments on grammatical errors throughout the manuscript. Overall, the paper is written in standard English in an intelligible fashion.

**Response:** Thank you for your overall positive evaluation of the manuscript.

**Reviewer 2 Comment 2 (all regarding grammatical errors):**

Sentence 54-55: COVID-19 is a pandemic respiratory disease [5, 6] that led many countries to order lockdowns that reduce social contacts.

*I think you can delete pandemic from this sentence, as it was mentioned in the previous sentence.*

Sentence 69: The following sentence could be made better with grammatical changes. “We argue that an important factor that is associated with well-being in the pandemic in general—and in life events that occur during the pandemic in particular—is perceived control.”

*Maybe instead you could say, “We argue that an important factor associated with general well-being during the pandemic, and with particular life events that occur during the pandemic, is perceived control.*

Sentence 76: “alongside the significant life event” – *perhaps change to a significant life event*

Sentence 135: “Data were collected online between June 23 and July 8, 2020.” -- *Change were to was, or data were to data points were.*

Sentence 205: “fit according to Cohen.” -- *Perhaps specify Cohen’s d*.

Sentence 215: “life-event experience, there were a significant association”. – *There were significant associations*

**Response:** Thank you for these recommendations. We have implemented them in the text.

**Reviewer 2 Comment 3:**

Did your lab collect data on self-reported anxiety and/or depression? As mentioned, in sentence 94-95, research had found the pandemic had a deleterious effect on adult mental health. The two variables would seemingly have important implications for individual perception of perceived control and the valence of life events. If you did collect measures of depression and/or anxiety, I would consider controlling for these two variables and rerunning your analyses. If not, I think adding a note of this in the limitations would be worthwhile.

**Response**: Thank you for this suggestion. Unfortunately, we did not collect data on anxiety and/or depression. We included this limitation in the discussion (L346-350).

**Reviewer 2 Comment 4:**

What is the justification for not controlling for gender?

**Response:** We conducted the analyses with gender and education as control variables (see supporting information material). Including these two variables in the analyses resulted in no systematic changes in the results. Thus, we omitted gender and education as control variables from the analyses. We report it in the manuscript (L121-126).

**Reviewer 2 Comment 5:**

Did you collect data on socioeconomic status? Differences in income would seemingly be important to understand in how they may explain the relationship between perception of positive or negative influence of the pandemic on major life events and perceived self-control. Income is an additional variable I would consider controlling for if you did collect data for this measure.

**Response:** We did not collect data on socioeconomic status. However, as education highly correlates with socioeconomic status, it can be used as an indicator of socioeconomic status (see also White, 1982; Sirin, 2005). Controlling for education did not substantially change the results (see our response to your comment #4).

**Reviewer 2 Comment 6:**

It may be worth noting that the following results may look different based upon geographical location. The following results may differ in countries where social systems such as universal healthcare and economic aid during the pandemic differed.

**Response:** Thank you for this comment. Because we investigated the subjective impact of the pandemic, rather than the objective impact, our results should be generalizable across countries. Nevertheless, we have included this point in the discussion (L351-355).

**Reviewer 2 Comment 7:**

In sentence 57 you mention how ongoing stressors may lead to more negative responses to significant life events, although stress was not mentioned or controlled for in analyses. Did you include a measure of stress in your data collection process?

**Response:** We apologize for the confusion. We have changed the wording from “stressors“ to “worry” (L53) or deleted the wording completely. Stress per se was not assessed.

**Additional clarifications or changes:**

We have made blinded-for-review information visible. We have changed the formatting according to the journal requirements. We have deleted references from the reference list that were not mentioned in the text and added those that were new (see Reviewer 1 Comment #2 and Reviewer 1 Comment #11). Note that the formatting in the text and in the reference list is according to the requirements of the journal and may therefore differ from the APA manual. Captions for supporting information were added at the end of the manuscript.
