# Supplementary material for: Experiencing a significant life event during the COVID-19 pandemic: The role of perceived control
Source: PLoS One. 2023 Jan 4;18(1):e0279820. doi: 10.1371/journal.pone.0279820 (PMC9812308; doi:10.1371/journal.pone.0279820)
Supplement: Attachment — Submitted filename: Response to Reviewers.docx [file pone.0279820.s004.docx]

Dear Dr. Murakami,

We are grateful for the opportunity to revise our manuscript based on the helpful suggestions of the reviewers. Below we address all the questions raised by the reviewers. Thank you for considering our research for publication.

Sincerely,

The Authors

**Reviewer 3 Comment 1:**  General comments
This study is a cross-sectional study of an age heterogeneous sample recruited through an online campaign in German-speaking countries. The focus of the study is very interesting, as the study reveals the hypothesis testing that control perception is an important factor in whether the COVID-19 pandemic affects the experience of important life events. However, the methodology for the purpose of this study is deficient and the research framework needs to be modified.

**Response:** Thank you for the positive feedback on the relevance of our study.

**Reviewer 3 Comment 2:** a) Maior
Although this study purports to examine whether the pandemic affected life events, there is no comparison with data from non-pandemic periods, so the pandemic is only a
conditions only. In other words, at this point, the framework of the hypothesis may be that control cognition is a useful factor that influences the experience of life events during a pandemic period.

**Response:** We agree that a comparison group would have deepened our understanding of the life event experience during the pandemic (we had mentioned this in Discussion, L365-370) and had it been technically possible, we would have included a comparison group. To address this limitation from the outset, we asked participants directly about the impact of the pandemic on the life event. This allowed us to test the extent to which control over the life event could explain the relationship between self-reported influence of the pandemic on the life event and the experience of the life event. Of course, we cannot rule out the possibility that this may be true for other external factors, not just the pandemic. However, we believe that this possibility does not diminish our findings. Accordingly, we argue that the control perception is one exploratory mechanism for the impact of the pandemic on the experience of significant life events (L82-84). And we argue that people who are in control of the life event would experience it more positively, irrespective of age (L95-96).

**Reviewer 3 Comment 3:** a) Maior
Many scales are used, including outcomes, but none are standardized and there is insufficient explanation and citation of self-development scales. Many of the scales are 7 scales, but please explain in detail how cutoffs are established and aggregated. Reliability can be confirmed, but there is no validity verification and no standard of scientific evidence is apparent.

**Response:** We apologize for the incomplete information. We have now added the information on the life-event experience scale (L156-158) and perceived control scale (L162,163). Because the pandemic was relatively recent at the time of the study, scales for the self-reported influence of the pandemic on the life event were not available. Regarding the response scales: Please note that since we do not use cut-offs but test relationships between variables, the format of the response scales is not fixed. For our type of research questions, 4- to 7-point response scales are recommended to ensure sufficient reliability and validity (see Lozano et al., 2008).

**Reviewer 3 Comment 4:** b) Minor
Since it is difficult to understand the attributes of the subjects of the analysis, the authors should add a table of basic attributes, socioeconomic indicators, and other variables.

**Response:** Thank you for your suggestion. We included age, gender, education (as an indicator of socioeconomic status; see White, 1982; Sirin, 2005), and place of residence in the descriptive analyses (L206-213).

**Reviewer 3 Comment 5:**

Also, you would do well to add a diagram of the conceptual framework of this study to make it easier to understand.

**Response:** We understand that the framework is difficult to understand without a graphic illustration. We did so in Figure 2 in L309-314, but have now placed it more prominently where we report the hypotheses, and changed the wording slightly for better understanding (L115-116).

**Reviewer 3 Comment 6:** b) Minor

You exclude gender and education from your analysis because these factors do not affect your results, but life events such as changing jobs or retiring may affect them, so do you need to exclude them from your analysis?

**Response:** Although the results did not change significantly after controlling for gender and education, we have included the analyses with these control variables in the manuscript and slightly changed the wording or the results in the manuscript (L183-185, 191, 241, 242, 244, 246, 253, 265, 271, 282 - 287, 292, 293, 296, 302-304, 314, 605-622). In addition, we have now included the analyses without gender and education in the supporting information material (the tables that were previously included in the manuscript). We have revised Figures 1 and 2 as we have now included the control variables (L249-253 + L309-314). We added another table (S6 Table: mediation analyses without gender and education as covariates) in the supporting information (L617-622).

**Reviewer 3 Comment 7:**

You should describe in the method the contents of lines 114 through 124. Similarly, lines 124 through 125 are the contents of the result.

**Response:** As suggested, we have moved the contents of the lines mentioned to the methods under “Data-Analytical Strategy” (L178-185).

**Reviewer 3 Comment 8:**

You should describe the characteristics of the elderly who participate through the online campaign, since you explore the possibility of age effects in the introduction and specifically detail the negative impact of the pandemic on the elderly. Also, in line 94, you state that you would like to shift the focus from age differences to people's life circumstances, but it would be better to state that you are focusing on both age and life circumstances.

**Response:** We have added some more information to compare our sample with the general population (L213-217 + L542-550). Regarding your second comment, we agree that we test age as well as life circumstances. Our previous argument referred to the heterogeneous and inconsistent results on the role of age for the experience of life events in the previous literature, which suggests that other factors are more relevant. For this reason, we included the following sentence: “we argue that people who are in control of the situation would experience the life event more positively, irrespective of age”.

**Reviewer 3 Comment 9:**

In line 326, you state "older ages was associated with lower perceived control over the life events", but I am not sure which result you are referring to as the basis for this statement.

**Response:** For a better understanding of the discussed results, we have now added the hypotheses in parentheses after each result (L319, 322, 325, 331, 336).

**Reviewer 3 Comment 10:**
For the results of the hierarchical multiple regression analysis in Table 3/Table 4, you should preferably not discuss them collectively as life events, but also discuss the results for each characteristic event.

**Response:** Thank you for this comment. We have now added the discussion of the results of individual life events to the Discussion (L339-361).

**Reviewer 4 Comment 1:**

I’ve read the present study with great interest in the timely and important topic during the pandemic. The authors have addressed most of the concerns of the reviewers, but I would like to raise a few points that I believe the manuscript could improve further.

**Response:** Thank you very much for your positive evaluation and the changes you recommended.

**Reviewer 4 Comment 2:** 
Lines 51-63: I recommend adding a bit of the COVID-19 epidemic situation or the national response to the pandemic in the study area. Because the study focuses on the COVID-19 influence and the perceived control, the social background would help to understand the study background.

**Response:** Thank you for your suggestion. Reactions to the pandemic and the imposed rules were very heterogeneous within the German-speaking countries of the present study. We now point out this heterogeneity with some examples and explain that it was one of the reasons to ask the participants for their subjective assessment of the influence of the pandemic on the life event instead of the objective circumstances (see L383-396).

**Reviewer 4 Comment 3:**

Lines 105-113: Please state the aim of the study.

**Response:** We have now stated the aim of the study in L105-107.

**Reviewer 4 Comment 4:**
Lines 114-148: I think this paragraph should be written in the Methods.

**Response:** Thank you for your suggestion. We have moved the paragraph to “Data-Analytical Strategy” (L178-185).

**Reviewer 4 Comment 5:**
Lines 147-148: Did the authors use the term ‘subsample’ here for the present study sample (N= 882)? There is the other ‘subsample’ in line 200 and the legend of Table 2. I think the former two ‘subsamples’, who are the participants of this study, excluding those who had out-of-period events from the total of 6,688 subjects, may not be considered a subsample. In the present study, the number of the participants for overall analysis was 882, and for the analysis of seven clusters of life events (a subsample?) was 667.

**Response:** Thank you for pointing out this confusion. You are correct: a total of 6’688 participants completed the questionnaire, but only the pandemic sample of 882 participants was used for the overall analyses. The 667 participants are the subsample for the analyses on each significant life event included based on the power analysis. We have now changed the wording from “subsample” to “present-study sample” (L137, 138 + L206, 207).

**Reviewer 4 Comment 6:**
Line 175: I cannot understand ‘the six items.’

**Response:** Thank you for noticing this. We agree that the text was difficult to understand. We have changed the wording (L166-168).

**Reviewer 4 Comment 7:**

Data-Analytical Strategy,
Please clarify the regression model. It would be easier to understand the results section if you explained the age-adjusted model and how to evaluate its impact.

**Response:** Thank you for your suggestion. For the explanation of the regression analyses, see the Data-Analytical Strategy (see L174-185) and the notes to the Tables 3 and 4.

**Reviewer 4 Comment 8:**

Please explain how the age and influence intensity were treated as control variables (e.g. per decade or a continuous variable).

**Response:** We now report that both influence intensity (L170, 171) and age (L174, 175) are continuous variables.

**Reviewer 4 Comment 9:**
Please explain how the authors did exploratory analyses. For this point, the paragraph, Lines 287-292, might be available and should be placed in the Data-Analytical Strategy.

**Response:** We have moved the paragraph to Data-Analytical Strategy according to your suggestion (L186-191).

**Reviewer 4 Comment 10:**
The authors did not control for gender. To strengthen the reason for that, please explain the specific index (i.e. not ‘no systematic changes’ but not changes in regression coefficients or delta R2). I also recommend including gender in the Descriptive statistics (Supplementary Table 2).

**Response:** Thank you for your suggestion. See our response to Reviewer’s 3 Comment 6.

**Reviewer 4 Comment 11:**

Table 3: Are the βs for the positive/negative influence and perceived control the regression coefficients in the age and overall influence intensity-adjusted model or in the overall influence intensity-adjusted model (not adjusted by age)? Please clarify.

**Response:** Yes, the betas already include control for covariates. This is noted in the note to Table 3 (now also with gender and education).

**Reviewer 4 Comment 12:**
The authors could discuss the relationship between the study results and cultural or social characteristics in the study area (Europe) to help understanding the mechanism of the results.

**Response:** Thank you for your suggestion. We now discuss the role of perceived control as a function of the type of culture (individualistic vs. collectivistic) (see L397-406).
