# Supplementary material for: Experiencing a significant life event during the COVID-19 pandemic: The role of perceived control
Source: PLoS One. 2023 Jan 4;18(1):e0279820. doi: 10.1371/journal.pone.0279820 (PMC9812308; doi:10.1371/journal.pone.0279820)
Supplement: Attachment — Submitted filename: Response to Reviewers.docx [file pone.0279820.s005.docx]

Dear Dr. Murakami,

We are grateful for the opportunity to revise our manuscript based on your helpful suggestions that we address below. Thank you for considering our research for publication.

Sincerely,

Sonja Radjenovic, Christina Ristl, and Jana Nikitin

**Journal Requirements:**

Please review your reference list to ensure that it is complete and correct. If you have cited papers that have been retracted, please include the rationale for doing so in the manuscript text or remove these references and replace them with relevant current references. Any changes to the reference list should be mentioned in the rebuttal letter that accompanies your revised manuscript. If you need to cite a retracted article, indicate the article’s retracted status in the References list and also include a citation and full reference for the retraction notice.

**Response:** Thank you for the reminder. We reviewed the references list on completeness and accuracy. All the mentioned references in the manuscript can be found in the reference list and vice versa. To our best knowledge, none of them have been retracted. As a result of the reference revision, we adapted the references concerning website citations to the journal’s citation style (L428-430; L546-553; L578-581).

**Additional Editor Comments:**

**Comment 1:** Figure 2 is mentioned in the text before Figure 1. Please renumber the figures.

**Response:** Thank you for noticing this. We mentioned Figure 2 in the introduction to better illustrate the conceptual framework of the study. However, Figure 2 shows the results of the exploratory analyses that follow the main analyses depicted in Figure 1. Therefore, we decided to keep the order of the figures as it is consistent with the order of the analyses. Instead, we have now deleted the mention of Figure 2 in the introduction (L115).

**Comment 2:** For Figure 1, each plot probably represents more than one participant, because of the same value among the participants. Please use a bubble plot to show the number of participants.

**Response:** Thank you for your suggestion. We changed the figure now to a bubble plot (see as a separate file Fig1.tif), changed the order of the individual plots and the names of individual plot labels (A to F) according to the text in the manuscript, and added further explanation to the figure notes (L252).

**Comment 3:** There is an insufficient description to the limitations of the bias due to older people participating in the online campaign (older people in this study are more likely to have characteristics that make them more familiar with online surveys than older people in general).

**Response:** Thank you for pointing this out. We now discuss this on lines L371-374.

**No comments by the reviewers**

**Additional corrections:**

Deleted spaces between two words (L210, L519), deleted the word “exactly” (L217), changed the language format from German to English (USA) in Table 2 (L231) and 3 (L324)
